# Critical Care Cardiology Fellowship Training in China: Past, Present, and Future

**DOI:** 10.31083/RCM47987

**Published:** 2026-02-03

**Authors:** Wen-Jun Liu, Zhe Luo, Ming-hao Luo, Guo-wei Tu

**Affiliations:** ^1^Cardiac Intensive Care Center, Zhongshan Hospital Fudan University, 200032 Shanghai, China; ^2^Shanghai Key Laboratory of Lung Inflammation and Injury, Zhongshan Hospital Fudan University, 20032 Shanghai, China; ^3^Department of Anaesthetic and Critical Care, Harefield Hospital, Heart, Lung and Critical Care Group, Guy's and St Thomas' NHS Foundation Trust, UB9 6JH London, UK

Critical Care Cardiology (CCC) is a well-established global subspecialty with structured training pathways in many developed countries. This article explores China’s journey toward a standardized CCC fellowship, its historical context, current challenges, and strategic future directions.

## 1. The Past: Learning by Doing

The evolution of cardiac critical care in China has broadly mirrored international trends, beginning with the establishment of Coronary Care Units (CCUs) focused on the management of acute myocardial infarction. Over the past three decades, Chinese Cardiac Intensive Care Units (CICUs) have undergone a profound transformation, now providing advanced management for cardiogenic shock, end-stage heart failure, and patients requiring mechanical circulatory support [1, 2, 3, 4].

However, physician training has not kept pace with these rapid clinical requirements. Early CCC education was largely apprenticeship-based, with physicians transitioning from cardiology or general critical care residencies and acquiring specialized skills informally through hands-on experience. While this experiential model produced competent clinicians, it also resulted in heterogeneity in competence across institutions. The absence of standardized curricula, defined competency frameworks, or national certification mechanisms meant that the quality of care depended heavily on local expertise and individual mentorship rather than consistent national standards.

## 2. The Present: Progress and Persistent Gaps

Today, CCC training in China stands at a transitional stage. The national implementation of standardized fellowship programs in several disciplines, including general critical care medicine, represents a major step forward. Yet, CCC has not been formally recognized as an independent subspecialty with a dedicated training pathway.

This structural deficiency manifests in current training arrangements. For example, fellows in Internal Medicine–Critical Care may rotate through cardiothoracic surgery, while Surgical–Critical Care fellows may spend limited time on general cardiology wards. Few programs, however, guarantee structured, supervised exposure to high-acuity CICUs, creating a persistent gap between the competencies required in modern CICU practice and those developed during training.

Additional challenges include a shortage of trained CCC educators, reliance on traditional teaching methods, and limited use of new teaching methods [5, 6, 7]. Equally concerning is the inadequate emphasis on non-technical skills, such as interdisciplinary leadership, crisis communication, and end-of-life care, which are essential for safe and compassionate practice [8, 9, 10]. The lack of national certification further weakens professional identity and public confidence in the uniform competence of CCC specialists.

## 3. The Future: A Strategic Framework for Advancement

Bridging the gap between clinical complexity and educational infrastructure will require a coordinated, national strategy aimed at building a competency-based, multidisciplinary CCC training system.

### 3.1 To Establish a National Competency-Based Curriculum

**Fig. 1.**
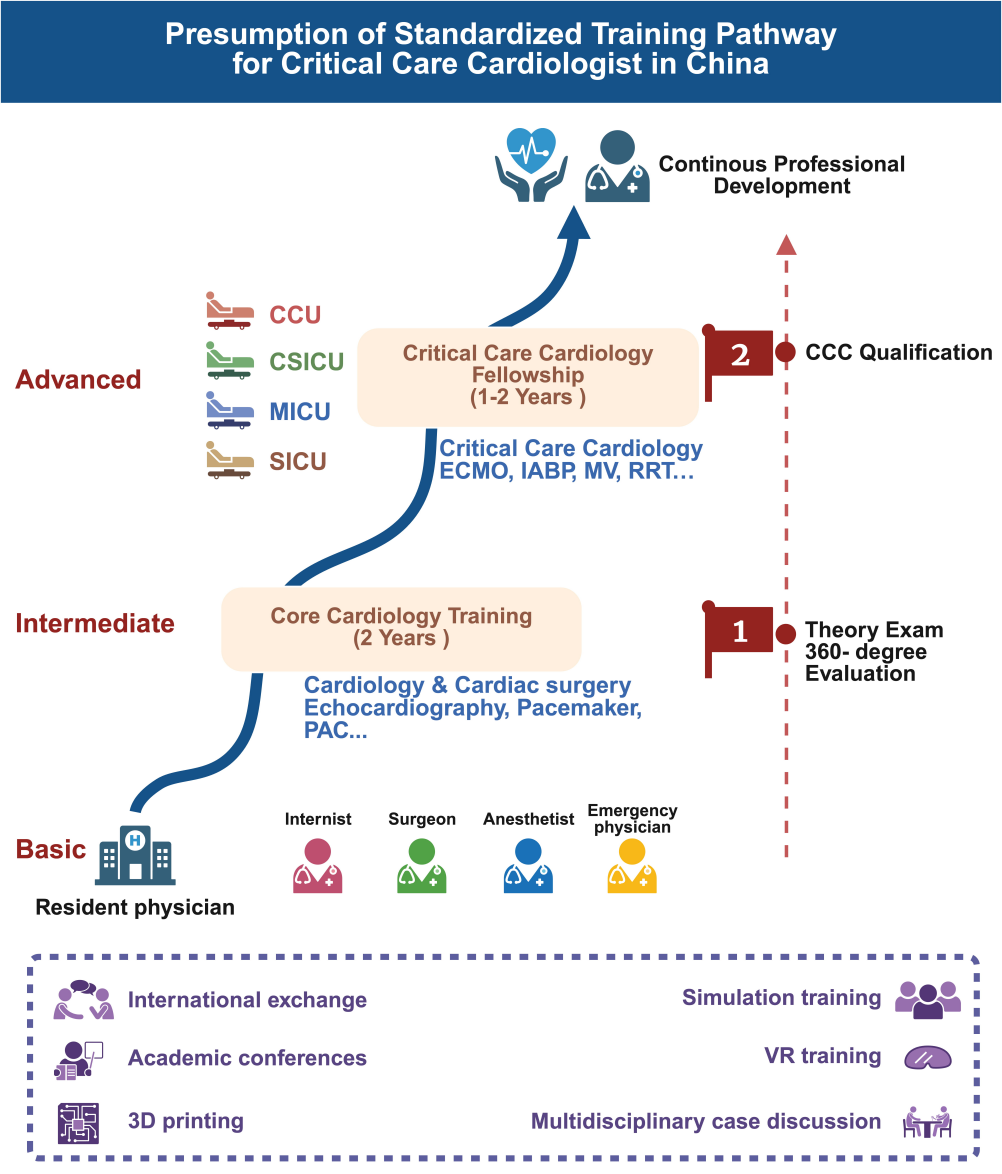
**Presumption of Standardized training pathway for critical care cardiologist in China**. CCU, coronary care unit; CSICU, cardiac surgery intensive care unit; MICU, medical intensive care unit; SICU, surgical intensive care unit; ECMO, extracorporeal membrane oxygenation; IABP, intra-aortic balloon pump; MV, mechanical ventilation; RRT, renal replacement therapy; CCC, critical care cardiologist; PAC, pulmonary artery catheter; 3D, three-dimensional; VR, virtual reality. Figure creation tool: https://www.biorender.com/.

A joint task force comprising experts from cardiology, critical care, cardiac surgery, and medical education should define the core knowledge domains, procedural competencies, and professional attributes expected of CCC physicians [11, 12]. This framework should specify longitudinal CICU rotations, required clinical exposures, and essential procedural skills [8, 13, 14]. We propose a 3–4 year training program aligned with China’s standard model and international benchmarks, structured as a “2+X” framework. This consists of two years of core cardiology rotations followed by 1–2 years of specialized intensive care unit (ICU) training in critical care and procedures. Physicians completing initial training in internal medicine, surgery, anesthesiology, or emergency medicine would undertake this pathway. During the core cardiology training, trainees will receive instruction in cardiology (including coronary care, electrophysiology, and structural interventions), cardiac surgery, and related disciplines (such as electrocardiography and cardiac anesthesia). During the critical care cardiology fellowship training, fellows will receive intensive care-related training. The first year involves rotations across various ICUs, aimed at mastering core competencies in critically ill patient management. The second year is optional, focusing primarily on cardiac ICUs with an emphasis on advanced critical care management and mechanical circulatory support (Fig. 1). A two-step implantation approach will be employed to facilitate the transition. In the first phase, a training framework based on rotation duration and case volume will be primarily adopted, and a competency-based training principle will be added in the second phase.

Regional hospitals may lack comprehensive departments, case diversity, or surgical capabilities; their physicians can therefore undertake delegated training at tertiary hospitals to gain the required exposure.

### 3.2 To Invest in Educator Development and Simulation-Based Teaching

Faculty development programs should be prioritized to train clinician-educators in modern pedagogical methods. “Train-the-trainer” initiatives and academic incentives (including protected teaching time, promotion criteria, funding and scholarship, and bonus system) for teaching excellence can cultivate a cadre of qualified mentors. Integrating simulation-based learning, Three-Dimensional (3D) printing, hybrid training, virtual and augmented reality, and scenario-based assessments will enhance procedural competency and crisis management skills in a safe learning environment [5, 6, 7, 10].

### 3.3 To Integrate Communication, Ethics, and Leadership Into the Curriculum

Structured modules on code leadership, family communication, and ethical decision-making should be included in the training [8, 9]. Assessment should move beyond written examinations toward 360-degree evaluations and direct observation of clinical performance, fostering both clinical excellence and emotional resilience [13, 14]. For instance, the Mini Clinical Evaluation Exercise (Mini-CEX) assesses brief clinical encounters (e.g., history-taking), while Direct Observation of Procedural Skills (DOPS) evaluates procedural skills (e.g., venipuncture). Regular peer assessments are conducted alongside patient experience feedback, which is collected through structured questionnaires.

### 3.4 To Foster International Collaboration and Exchange

Partnerships with established CCC programs internationally can accelerate curriculum development and faculty training [9, 11]. Joint workshops, visiting fellowships, and co-developed educational materials can facilitate mutual learning and empower China to integrate global best practices while avoiding known pitfalls.

## 4. Conclusion

The evolution of CCC in China from an experience-based discipline to a structured, competency-driven subspecialty is both an urgent need and a historic opportunity. Future reforms should emphasize standardized competencies, evidence-based educational methods, and integration of communication and ethical training. By developing a unified national training framework that includes both technical expertise and humanistic excellence, China can empower the next generation of cardiac intensivists to deliver high-quality, compassionate care to patients with critical cardiovascular disease, fulfilling the promise of modern CCC.
